# ALK 1 Negative Inflammatory Myofibroblastic Tumor of the Ileum: A Rare Cause of Ileocecal Intussusception

**DOI:** 10.1055/s-0040-1710531

**Published:** 2020-05-19

**Authors:** Vipul D. Yagnik

**Affiliations:** 1 Department of Surgical Gastroenterology, Nishtha Surgical Hospital and Research Centre, Patan, Gujarat, India

**Keywords:** inflammatory myofibroblastic tumor, small bowel, intussusception

## Abstract

An inflammatory myofibroblastic tumor is a rare tumor of mesenchymal background commonly found in the pulmonary system. It is rarely found as a primary tumor in the gastrointestinal tract. We report an unusual presentation of this rare lesion causing intussusception and intestinal obstruction in a 39-year-old male.

Inflammatory myofibroblastic tumor (IMT) belongs to a class of rare spindle cell tumors and was previously called inflammatory pseudotumor or plasma cell granuloma. The World Health Organization classifies IMTs as tumors of intermediate biological potential since both local recurrence and metastases are possible. 1 2 IMT affects children and young adults predominantly, but patients of any age and sex can be affected. 3 Gastrointestinal tract (GIT) IMT presents with clinical symptoms of anemia, loss of appetite or weight, fecal blood positive, abdominal pain, GIT obstruction, or intussusception. IMT is generally expected to have a benign course. However, extrapulmonary origin, size >8 cm, and local invasion are the factors associated with an elevated risk of recurrence which mostly occurs within 2 years of the initial surgery.

## Case History

A 39-year-old male presented to the surgical outpatient department with complaints of abdominal distension and pain for 3 to 4 days. He also reported vomiting and obstipation for the same duration. His past medical and surgical history was not significant. Physical examination revealed abdominal distension with tenderness in the right lower quadrant. Bowel sounds were hyperperistaltic. Laboratory investigations were normal. Abdominal ultrasound revealed bowel-within-bowel appearance in the right lower quadrant, the segment being approximately 8-cm long with a lead point of approximately 50 × 29 × 28 mm in the cecum.

**Fig. 1:**
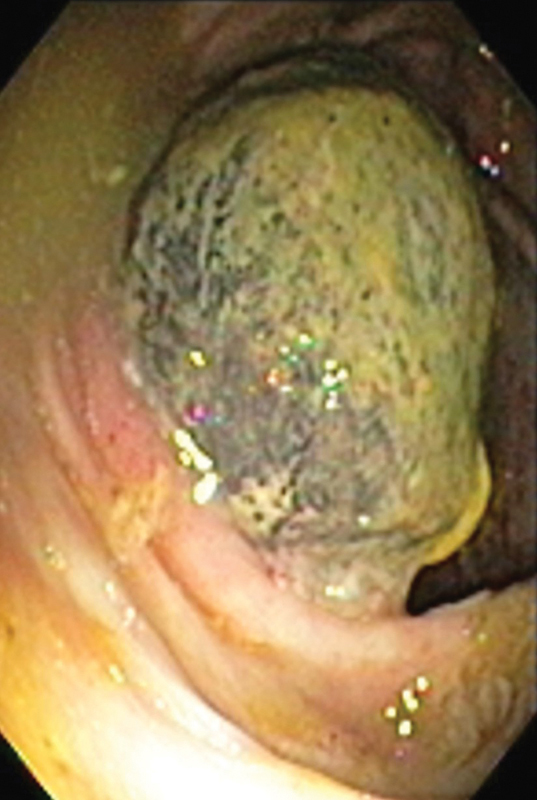
Colonoscopic image showing lead point projecting through ileocecal valve.

**Fig. 2:**
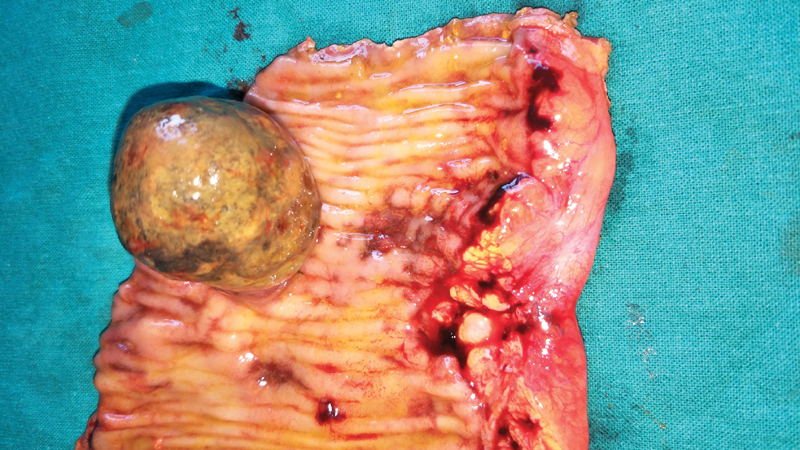
Resected segment of the small bowel showed polypoidal lesion.

**Fig. 3:**
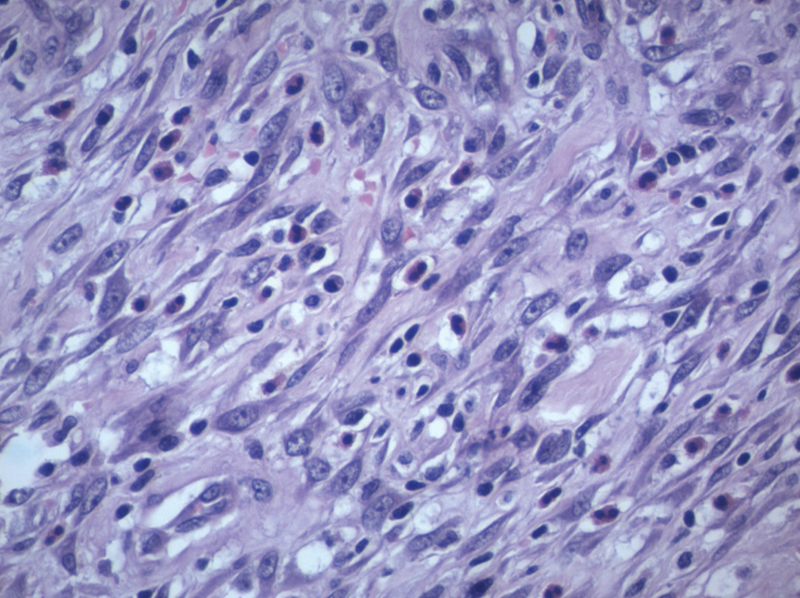
40X H& E stain: IMT composed of spindle cells along with mixed inflammatory infiltrate. H & E stain, hematoxylin and eosin stain; IMT, Inflammatory myofibroblastic tumor.

**Fig. 4:**
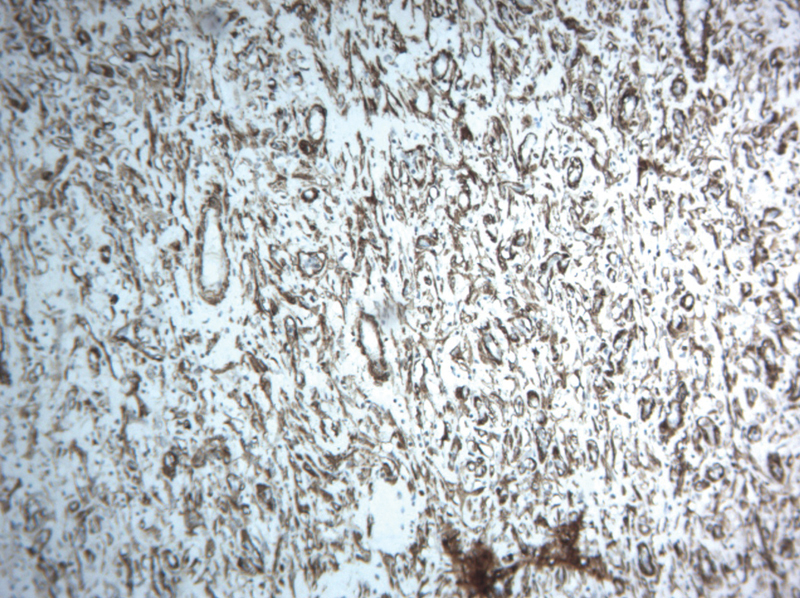
IHC 10X: IMT composed of spindle cells with SMA positive. IHC, immunohistochemistry; IMT, Inflammatory myofibroblastic tumor.

**Fig. 5:**
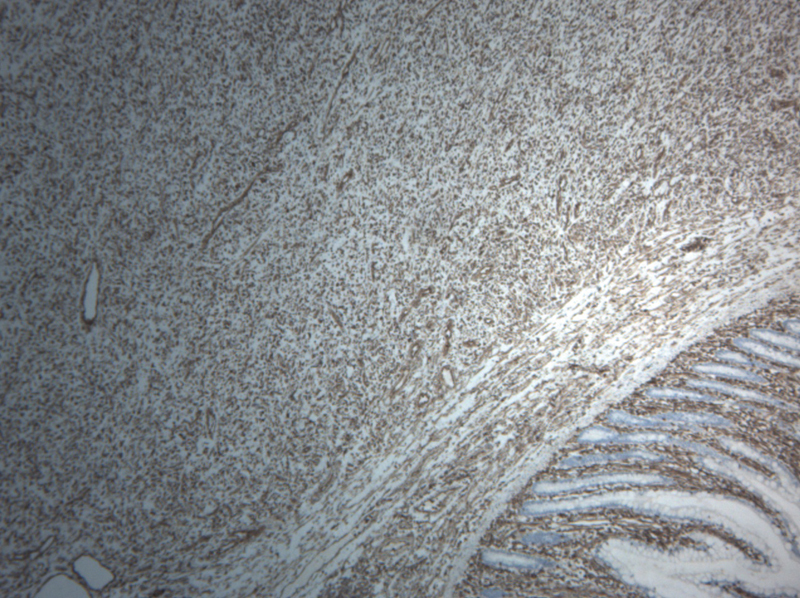
IHC 10X: IMT composed of spindle cells with Vimentin positive. IHC, immunohistochemistry; IMT, Inflammatory myofibroblastic tumor.

We performed colonoscopy and planned colonoscopic reduction and removal of the lead point. A lead point was seen projecting through the ileocecal valve ( Fig. 1 ). Though colonoscopic reduction was achieved, the lead point was beyond the limit of the standard length of the colonoscope. Therefore, we performed laparotomy via a midline incision. The lead point was palpated 25 cm proximal to the ileocecal junction. Segmental resection of the terminal ileum was performed. The resected segment revealed a polypoidal lesion ( Fig. 2 ). Histopathology of the specimen was consistent with a spindle cell tumor ( Fig. 3 ). Immunohistochemistry was positive for smooth muscle actin ( Fig. 4 ) and vimentin ( Fig. 5 ), but negative for CD117, DOG1, Desmin, CD34, S100, and ALK 1. Overall, the immunomorphological profile excluded gastrointestinal (GI) stromal tumors and suggested the diagnosis of IMT. The postoperative course was unremarkable and the patient was discharged on the fifth postoperative day. The patient has been followed up for 2 years without any problem.

## Discussion

An IMT is a rare tumor of mesenchymal background commonly found in the pulmonary system. Dr. H. Brunn first described IMTs in 1939. 4 It is also known as a pseudotumor with malignant potential. 5 The World Health Organization classifies IMT as tumors of intermediate biological potential since both local recurrence and metastases are possible. 1 2 Coffin et al 3 showed that IMT developed at a mean age of 9.7 years, and in 36 of 84 cases (43%), IMTs arose from the mesentery and omentum. Höhne et al 6 reviewed 443 publications from between 2009 to February 2014; their reports involved 938 patients and 956 organ sites. They found liver involvement in 31.8% of the cases followed by lung involvement in 18.2% cases. Abdominal association excluding the liver was noted in 15.5% of the cases. Out of 956 organ sites affected, involvement of the small bowel was reported in only 11 locations (1.15%). 6 GIT IMT presents with clinical symptoms of anemia, loss of appetite or weight, fecal blood positive, 7 abdominal pain, GI obstruction, 8 or intussusception. 9 Immunohistochemistry helps to differentiate IMT from other spindle cell tumor-like GI stromal tumors, leiomyosarcomas, and inflammatory malignant fibrous histiocytomas. 10 Surgical excision is considered the treatment of choice for a GIT IMT, although anti-inflammatory drugs and/or chemoradiotherapy have been used. 11 12 Approximately 50% of the IMTs have a clonal rearrangement of the ALK gene and this can also be used as a specific marker to differentiate other tumors. However, ALK negativity is not a criterion to exclude IMT. ALK positivity is seen more in younger individuals and is associated with a higher recurrence rate. 11 Extrapulmonary origin, size >8 cm, and local invasion are the factors associated with an elevated risk of recurrence. 13 Postsurgical adjuvant treatments might be considered in abdominal IMT as it has the highest rate of local recurrence (25%). 14 Long-term follow-up is advisable in the patient treated with surgery to investigate the risk of recurrence. 15 16

## Conclusion

Intussusception due to IMT is rare. Diagnosis of IMT involving the GIT requires a high index of suspicion, detailed history, physical examination, and imaging studies that are necessary for early recognition and diagnosis. Surgery is the cornerstone of treatment. Long-term follow-up is required to detect recurrence.
